# Proceedings of the National Medical Convention

**Published:** 1847-08

**Authors:** 


PROCEEDINGS OF THE NATIONAL MEDICAL CONVENTION.

The proceedings of both Conventions—that which sat in New York, and that recently held in Philadelphia—are given in this report, of which a copy has been for some days on our table. We have transferred to our pages one of the most interesting of the reports adopted by the Convention and given in the Appendix to this volume—that on MEDICAL ETHICS, and cannot too strongly recommend it to the perusal of our readers. Every physician in the land ought to be in possession of a copy of this report, and it would be well if the substance of it could be lodged in the mind of every man who may have occasion for the services of a physician. Ignorance or disregard of the observances insisted upon in this document leads to countless bickerings among the members of the profession, and if the labors of the Convention should result in nothing more than the general, honest adoption of this code, that will be an object worthy of the united efforts of the profession. It is due to Dr. Hays to make an explanation with which he accompanied the report. It will be remarked by the reader that the language in various passages is that with which he has met before in other codes of ethics. On examining a great number of such codes Dr. H. found that they were all based on that by Dr. Percival, and that the phrases of this writer were preserved, to a considerable extent, in all of them. He deemed it better to pursue a similar course, and has carefully preserved the words of Percival whenever they convey the precepts it was wished to inculcate. Some of the sections will be recognised as the precepts of Dr. Rush, and a few sentences are from other writers.

The third report in these proceedings is "on the standard of acquirements which should be exacted of young men before being received as students of medicine." The authors of this report recommend that, besides having a good moral character, every young man proposing to study medicine should have "a good English education, a knowledge of Natural Philosophy and the Elementary Mathematical Sciences, including Geometry and Algebra; and such an acquaintance, at least, with the Latin and Greek languages as will enable them to appreciate the technical language of medicine, and read and write prescriptions."

We shall not stop now to discuss this proposition. In the abstract, we see nothing in it particularly to object to. It is desirable that students of medicine should be prepared by some previous training, and the course recommended by the committee is probably not too extensive. But is to be borne in mind that not a few eminent physicians, themselves scholars, have declared against the utility of the Latin and Greek languages as a part of the education of medical men. Latin prescriptions, we may hope, in half a century more, will be universally abandoned, and so far as relates to reading and writing them the necessity of this tongue will not be urged. Still, there must be some preparatory course of study, if we are ever to see an educated faculty in our country, and the standard of the committee may as well be held up before the eyes of our medical youth. It may be a long time before the exaction can be successfully made, but it is time that something higher than the present requisitions should be thought of and attempted. The public mind must be enlightened touching the importance of an educated profession, and from the profession itself the light must emanate.

